# Diagnostic value and utility of commonly used biomarkers of cardiac and renal function in cardiorenal syndromes: a narrative review

**DOI:** 10.11613/BM.2023.030502

**Published:** 2023-08-05

**Authors:** Điđi Delalić, Tanja Brežni, Ingrid Prkačin

**Affiliations:** 1 University of Zagreb School of Medicine, Zagreb, Croatia; 2 Faculty of Pharmacy and Biochemistry, University of Zagreb, Zagreb, Croatia; 3 Emergency Internal Medicine Clinic, Clinical Hospital Merkur, Zagreb, Croatia

**Keywords:** acute kidney injury, biomarkers, cardiorenal syndrome, heart failure, renal insufficiency

## Abstract

Cardiorenal syndrome (CRS), first defined in 2004 as a consequence of the interactions between the kidneys and other circulatory departments leading to acute heart failure, has since been recognized as a complex clinical entity that is hard to define, diagnose and classify. The framework for the classification of CRS according to pathophysiologic background was laid out in 2008, dividing CRS into five distinct phenotypes. However, determining the timing of individual organ injuries and making a diagnosis of either renal or cardiac failure remains an elusive task. In clinical practice, the diagnosis and phenotyping of CRS is mostly based on using laboratory biomarkers in order to directly or indirectly estimate the degree of end-organ functional decline. Therefore, a well-educated clinician should be aware of the effects that the reduction of renal and cardiac function has on the diagnostic and predictive value and properties of the most commonly used biomarkers (*e.g.* troponins, N-terminal pro-brain natriuretic peptide, serum creatinine *etc*). They should also be acquainted, on a basic level, with emerging biomarkers that are specific to either the degree of glomerular integrity (cystatin C) or tubular injury (neutrophil gelatinase-associated lipocalin). This narrative review aims to provide a scoping overview of the different roles that biomarkers play in both the diagnosis of CRS and the prognosis of the disease in patients who have been diagnosed with it, along with highlighting the most important pitfalls in their interpretation in the context of impaired renal and/or cardiac function.

## Defining cardiorenal syndrome

The pathophysiologic relationship between the heart and the kidney has been well known and studied throughout the history of modern medicine, however it was not until the early 2000s that an attempt was made to formally describe and classify the disruption of homeostasis arising from the interactions between these two organ systems. In 2004, the National Heart, Blood and Lung Institute established the term “cardiorenal dysregulation” and described it as a result of interactions between the kidneys and other circulatory compartments that increase circulatory volume, thereby exacerbating the degree of heart failure severity (*1*). In that context, cardiorenal syndrome (CRS) was defined as the extreme end of the cardiorenal dysregulation spectrum, a situation where efforts to relieve heart failure by stimulating diuresis are stifled by the decline in renal function. Several groups of authors and clinical societies have since modified the original definition of CRS. In 2008, the Acute Dialysis Quality Initiative (ADQI) made the distinction between cardiorenal and renocardiac syndromes, with the nomenclature being determined by the organ system of the primary disease process. The same group also divided CRS into five different phenotypes, based on the primary organ system affected and the chronicity of the disease process (*2*). The phenotypes are outlined in Table 1. Although these phenotypes have provided an abundant amount of utility to clinicians by providing them with a framework for classifying a wide spectrum of clinical entities into categories and deciding on the appropriate treatment course and modality based on the specific phenotype, there is often overlap between two different phenotypes. In addition, during the progression of the disease process, it is possible that one phenotype evolves into another, further complicating the diagnostic and treatment processes. Another layer of complexity is added by the fact that many patients suffer from comorbidities that further compromise both renal and cardiac function, such as arterial hypertension, anaemia, atherosclerosis and diabetes mellitus. An attempt to account for these factors was made by Hatamizadeh *et al*., suggesting a classification of CRS based on the predominant clinical symptoms and manifestations (*3*) (Table 2). Besides the disagreements on the basis for classification of CRS, another significant challenge for clinicians is making the distinction between chronic and acute kidney injury (AKI). Several different diagnostic criteria and tools have been described and established in everyday clinical use, with the most common ones being the Acute Kidney Injury Network (AKIN) criteria, ADQI’s Risk, Injury, Failure, Loss of Function, End Stage Renal Disease (RIFLE) criteria and the Kidney Disease: Improving Global Outcomes (KDIGO) criteria, which combined RIFLE and AKIN (*4*-*6*) (Table 3). As can be seen in Table 3, all of the listed criteria use serum creatinine and/or estimated glomerular filtration rate (eGFR) as components for determining the presence of AKI. However, there is evidence that temporary fluctuations in serum creatinine (and by extent, eGFR) in the context of the treatment of heart failure may not accurately reflect the structural and functional integrity of the kidney or serve as a reliable indicator of acute kidney injury (*7*). Therefore, it is of crucial importance to be acquainted with the biomarkers used for estimating cardiac and/or renal function, their prognostic and diagnostic utility, and indications for their use, in order to accurately diagnose, classify and treat CRS.

**Table 1:** Pathophysiology-based classification of cardiorenal syndrome

**Cardiorenal syndrome phenotype**	**Name**	**Pathophysiology**	**Example**
Type I	Acute cardiorenal syndrome	Heart failure leading to acute kidney injury	Acute decompensated heart failure leading to acute kidney injury
Type II	Chronic cardiorenal syndrome	Chronic heart failure leading to chronic kidney disease	Chronic heart failure
Type III	Acute renocardiac syndrome	Acute kidney injury leading to acute heart failure	Acute kidney injury leading to uraemia and volume overload, resulting in acute heart failure
Type IV	Chronic renocardiac syndrome	Chronic kidney disease leading to chronic heart failure	Heart failure and left ventricular hypertrophy as a result of cardiomyopathy associated with chronic kidney disease
Type V	Secondary cardiorenal syndrome	Systemic disease process leading to simultaneous renal injury and heart failure	Hepatic cirrhosis, sepsis
Adapted from Ronco *et al.* (2).			

**Table 2:** Classification of cardiorenal syndrome according to leading clinical presentation

**Class of cardiorenal syndrome**	**Description**
1 Haemodynamic	Haemodynamic compromise is the leading clinical manifestation
2 Uremic	Uremic manifestations are the leading clinical presentation
3 Vascular	Cardiovascular and/or renovascular manifestations are the leading clinical presentation
4 Neurohumoral	Electrolyte disorders, acid-base disorders or dysautonomia are the leading clinical manifestation
5 Anaemia/iron metabolism	Anaemia and/or iron metabolism dysregulation are the leading clinical manifestations
6 Mineral metabolism	Dysregulation of calcium and phosphorus and their regulators including vitamin D and fibroblast growth factor 23 (FGF23) are the leading clinical manifestations
7 Malnutrition/inflammation/cachexia	Malnutrition, cachexia and inflammation are the leading clinical manifestations
Adapted from Hatamizadeh *et al.* (3)	

**Table 3:** Most commonly used criteria for diagnosing acute kidney injury

**Criteria name**	**Components**
RIFLE	Dialysis for > 3 months
	Renal replacement therapy for > 4 weeks
	Serum creatinine increase relative to baseline (1.5x for risk, 2x for injury, 3x or creatinine ≥ 354 µmol/L with acute rise of ≥ 44 µmol/L for failure)
	Glomerular filtration rate decrease relative to baseline (> 25% for risk, > 50% for injury, > 75% for failure)
	Urine output (< 0.5 mL/kg/h through 6 hours for risk, < 0.5 mL/kg/h through 12 hours for injury, < 0.3 mL/kg/h through 24 hours or anuria for 12 hours for failure)
AKIN	Absolute increase in serum creatinine concentration > 26.4 µmol/L
	Increase in serum creatinine concentration > 1.5x relative to baseline
	Urine output < 0.5 mL/kg/h for > 6 hours
KDIGO	Serum creatinine
	Urine output
RIFLE – Risk, Injury, Failure, Loss of kidney function, and End-stage kidney disease, criteria first described by Bellomo *et al*. (5). AKIN – Acute Kidney Injury Network, criteria first described by Mehta *et al*. (4). KDIGO – Kidney Disease Improving Global Outcomes, criteria first described by the Kidney Disease: Improving Global Outcomes (KDIGO) Acute Kidney Injury Work Group (6).	

## Renal biomarkers in cardiorenal syndrome

### Creatinine

Serum creatinine is the most commonly used biomarker of renal function, applicable both for evaluating the progression of chronic kidney disease and the onset of acute kidney injury. It is the end product of creatine metabolism in the human body (*8*). Due to its low molecular weight and not being bound to albumin, creatinine is mostly eliminated through glomerular filtration, with a small amount secreted actively by the proximal renal tubules (evident by the fact that medication which block the secretion from proximal tubules increase the concentration of serum creatinine) (*9*). It has been proven that in patients with chronic kidney disease (CKD), clearance of creatinine by proximal tubular secretion increases as glomerular filtration decreases. Therefore, in patients with CKD, estimating glomerular function with creatinine clearance or serum creatinine concentration alone may actually lead to overestimation of the GFR (*10*). In the setting of acute kidney injury, aggressive intravenous fluid resuscitation may either decrease the serum creatinine concentration by causing dilution or increase it by causing venous congestion and increasing the renal interstitial pressure, making it an unreliable marker of actual renal function (*11*, *12*). Another important point to make is that estimating the GFR from a single serum creatinine measurement is a rather serious pitfall that should be avoided. A study of patients visiting the emergency department (ED) in The Netherlands showed that almost a third of the patients had a significant change (> 15% increase or > 18% decrease) in the measured serum creatinine when the value obtained in the ED was compared to either their baseline or the repeat value measured 6 to 24 hours following admission to the hospital ward (*13*). Caution should also be applied with acute heart failure patients - data obtained from the Diuretic Optimization Strategies Evaluation (DOSE) trial showed that an increase in serum creatinine concentration during the application of diuretic therapy was associated with lower rates of death and hospital readmission within 60 days, while a decrease in serum creatinine concentration (*i.e.* improvement in renal function) was associated with significantly higher rates of adverse outcomes within 60 days (*14*). It is important to note that while serum creatinine by itself may not be a reliable marker of AKI, some studies have demonstrated an increase in diagnostic accuracy when it is combined with other biomarkers. For example, one study found that a nomogram which combined serum creatinine concentration values measured on the first day of hospitalization and neutrophil gelatinase-associated lipocalin (NGAL) serum concentrations had an area-under-the-curve (AUC) of 0.79 for predicting the development of type I CRS, demonstrating a significantly higher diagnostic accuracy than serum creatinine alone (*15*). While there are several pitfalls to using creatinine as the principal biomarker of renal function and tissue integrity, it is necessary to note that calculations like the Modification of Diet in Renal Disease (MDRD) formula and the CKD Epidemiology Collaboration (CKD-EPI) formula, which are the ones most frequently used to estimate GFR, use the serum creatinine concentration as an essential part of their equations. A recent position statement by the European Federation of Clinical Chemistry and Laboratory Medicine (EFLM) recommended using the 2009 version of the CKD-EPI formula for the estimation of GFR, but also mentioned cystatin C-based equations as growing in popularity (*16*).

### Cystatin C

Cystatin C is a protein with an important role in the regulation of extracellular proteolysis (*17*). It is produced in the body at a constant rate, freely filtered through the glomeruli and reabsorbed at the brush border of the proximal renal tubules, where it is ultimately degraded. It is also independent of age, gender or muscle mass, unlike creatinine (*18*). Cystatin C is an important marker in CRS and it has been studied as a prognostic marker of both renal and cardiac outcomes. The findings of a meta-analysis of studies using cystatin C as a prognostic marker in patients with acute heart failure point to an increased risk of all-cause mortality (hazard ratio (HR) = 2.33; 95% confidence interval (CI): 1.67-3.27, P < 0.001) in patients with elevated serum cystatin C concentrations (*19*). The increased risk was observed in the subgroup analyses of the included studies and persisted regardless of heart failure type (acute *versus* chronic), study sample size or cystatin C cut-off value. A study by Pinsino *et al*. compared the accuracy of eGFR estimated with creatinine to eGFR estimated with cystatin C for predicting a composite outcome of in-hospital mortality, the need for renal replacement therapy or severe right ventricular outcome in patients with a recently implanted left ventricular assist device (LVAD) due to advanced heart failure. The authors found that eGFR estimated by cystatin C was significantly correlated to the composite outcome (odds ratio (OR) 1.16, 95%CI 1.02-1.31; for each 5mL/min/1.72m^2^ decrease in eGFR). No significant correlation with the primary endpoint was found with eGFR estimated with creatinine (*20*). A longitudinal Chinese study of more than 7000 participants found that increased serum cystatin C concentrations compared to baseline were significantly associated with the occurrence of new-onset cardiovascular disease (defined as newly diagnosed heart disease, stroke or both) (*21*). Cystatin C has also been studied as a predictor of outcomes in CRS in several studies, either by itself or combined with other biomarkers. Ruan *et al.* studied 162 patients with acute heart failure and found that increased cystatin C serum concentrations were significantly associated with increased 12-month mortality (OR 2.72, 95%CI 1.92-4.28, P = 0.017), while patients with AKI and an increased serum cystatin C had significantly higher rates of both in-hospital and 12-month mortality compared to patients without AKI (*22*). Rafouli-Stergiou *et al*. performed serial measurements of serum cystatin C in 96 patients with acute decompensated heart failure with an ejection fraction < 35% and creatinine clearance < 60mL/min and observed their post-discharge outcomes. They found that an in-hospital increase in serum cystatin C of ≥ 0.4 mg/L was an independent predictor of death or hospital readmission due to decompensated heart failure within 60 days (*23*). Cystatin C is also extremely valuable as an exclusively renal biomarker. The findings of a meta-analysis of 30 prospective studies with more than 4000 patients indicate that an increased serum cystatin C is significantly associated with all-cause AKI, with an AUC of 0.89, 82% sensitivity (95% CI 0.75-0.87), 82% specificity (95% CI 0.78-0.86), and a positive likelihood ratio of 4.6 (95% CI 3.6-5.9), making it a reliable marker of AKI (*24*). Studies have also been conducted to determine the value of cystatin C in chronic kidney disease classification. The Renal Risk in Derby (RRD) study examined 1741 primary care adult patients over the age of 70 with chronic kidney disease grade 3a or 3b determined by eGFR estimated from serum creatinine. Following serum cystatin C concentration measurements, 7.7% of patients were reclassified as not having CKD, while 59% were reclassified to a more advanced stage of CKD (*25*). In a study with younger patients (aged 55-60), 12% of patients with CKD G3a were reclassified as not having CKD and 6% were reclassified to a more advanced CKD stage (*26*). Regardless of age, it has been proven that cystatin C, when used together with creatinine to estimate eGFR, improves the strength of association of eGFR with adverse outcomes and is therefore a useful biomarker in improving the staging of CKD, especially in the cases where clinical suspicion of an inaccurate creatinine-based eGFR value exists (*25*, *26*). Summarily, cystatin C is a better marker of renal function in CKD than serum creatinine and can help clinicians appropriately and accurately determine the stage CKD in order to avoid over or undertreating patients. In the context of acute heart failure and CRS, elevated cystatin C values are a decent predictor of post-discharge outcomes and can serve as a valuable tool in stratifying patients based on risk of adverse post-discharge outcomes. This may assist clinicians in recognizing the patients who are at the greatest risk of repeat decompensation in order to provide more aggressive treatment and more frequent follow-ups.

### Neutrophil gelatinase-associated lipocalin

Neutrophil gelatinase-associated lipocalin (NGAL) is a member of the lipocalin family, a group of proteins in the human body serving mostly as transporters of other molecules (*27*). It is increasingly synthesized and expressed on the cells of the proximal and distal renal tubules following acute ischemia and is therefore a marker of acute renal tubular injury and necrosis (*28*). It has been investigated as a diagnostic and predictive factor in both acute kidney injury and CRS. Song *et al*. studied the association between type I CRS and serum NGAL concentration. They found that increased serum NGAL concentrations were an adequate diagnostic tool for type I CRS, with an AUC of 0.88 (95%CI 0.81-0.94), 95% sensitivity and 81% specificity. When combined with NT-proBNP, the AUC was 0.92 (95%CI 0.87-0.96), with a 93% sensitivity and 80% specificity (*29*). Another prospective study by Alvelos *et al*. demonstrated that above a cut-off concentration value of 170 ng/L, serum NGAL determined type I CRS with an AUC of 0.93 (95%CI 0.88-0.98), 100% sensitivity and 87% specificity (*30*). However, in a retrospective study of patients with type I CRS by Ferrari *et al.*, serum NGAL had an AUC of only 0.45 (95%CI 0.36-0.54) and its concentration was not significantly associated with the risk of developing CRS. The authors hypothesize that the result may be skewed due to the studied population suffering from relatively mild renal impairment and low-grade heart failure (*31*). Other studies have investigated the predictive value of NGAL for the development of AKI in acute heart failure patients. The AKINESIS study found that serum NGAL was not superior to serum creatinine for prediction of worsening of renal function or adverse in-hospital outcomes in patients with acute heart failure (*32*). Soyler *et al*. studied the utility of urinary NGAL for prediction of AKI in patients with acute decompensated heart failure and found that urinary NGAL concentrations above the cut-off value of 12 ng/mL were predictive of AKI with a sensitivity of 79% and a specificity of 67% (*33*). Another study conducted by Nasonova *et al.* showed an AUC of 0.83 with a sensitivity of 83% for urinary NGAL in the prediction of AKI in patients with acute decompensated heart failure (*34*). Palazzuoli *et al*. showed that serum NGAL concentrations above the cut-off value of 134 ng/mL are significantly related to worsening of renal function, with an AUC of 0.83, 92% sensitivity and 71% specificity. Serum NGAL concentrations above the cut-off value of 134 ng/mL also statistically significantly correlated with death (HR = 1.75, 95%CI 1.24-2.45, P < 0.001) (*35*). Maybe the most important and clinically useful characteristic of NGAL is its apparent ability to indicate subclinical AKI. Haase *et al*. found that in patients who had subclinical acute kidney injury with serum creatinine concentrations within reference intervals, but had increased concentrations of NGAL, there was a significantly increased risk of needing renal replacement therapy during hospitalization (OR 16.4, 95%CI 3.6-76.9, P < 0.001), with the results consistent regardless of using serum or urinary NGAL measurements (*36*). This feature of NGAL allows clinicians to make a timely diagnosis of AKI and initiate proper preventive and curative measures in time to prevent further short and long-term renal function decline. Neutrophil gelatinase-associated lipocalin has also been demonstrated as a reliable diagnostic biomarker of AKI within the clinical context of systemic inflammation in critical illness, accurately predicting the development of AKI in patients with sepsis regardless of its severity (categorized by serum procalcitonin concentration) (*37*).

In summary, literature on NGAL as a diagnostic biomarker of type I CRS features a certain degree of heterogeneity in findings and results. Some studies have found excellent sensitivities (> 90%) and satisfactory specificities (> 80%), indicating NGAL as a useful marker for both including and excluding the diagnosis of type I CRS (*29*, *30*). Others (*31*) have failed to demonstrate its utility and found its diagnostic accuracy to be grossly inadequate, although their results must be interpreted in the context of the study population featuring less severe clinical presentations and disease severity compared to the populations in other studies on the topic. The effects of the degree of severity of type I CRS on the diagnostic utility of NGAL should be further studied in order to find the patient and disease characteristics that point to no benefit of using NGAL as a diagnostic tool.

### Albuminuria

Albuminuria is an important marker of renal function and has been recognized as such for a long time. In the 2012 KDIGO classification of chronic kidney disease, CKD was divided into five grades numbered 1 through 5, with grade 3 being further divided into a and b subgrades based on eGFR and into three stages based on the degree of albuminuria calculated using the albumin/creatinine ratio (*38*). The presence of albuminuria as an essential factor in staging the severity of CKD is the result of several large population studies showing strong significant correlation between albuminuria and all-cause mortality, cardiovascular disease, and end stage renal disease (*39*-*41*). Recent *in vitro* studies of a type I CRS model using human kidney cells have demonstrated that albumin damages renal tubules in a dose-dependent fashion, thereby implicating it as an important pathophysiological factor further exacerbating AKI in experimental conditions (*42*). Albuminuria has been studied as a disease prognosis marker in both acute and chronic heart failure as well. A study by Jackson *et al.* investigated the association between albuminuria and outcomes in chronic heart failure and found a significant association between both microalbuminuria and macroalbuminuria and all-cause mortality (for microalbuminuria: HR = 1.62, 95%CI 1.32-1.99, P < 0.001; for macroalbuminuria: HR = 1.76, 95%CI 1.32-2.35, P = 0.001) after adjusting for diabetes mellitus, renal function and glycated haemoglobin (HbA1c) (*43*). A study of cardiac morphology in patients with CKD by Landler *et al.* found more than a 4-fold increase in the prevalence of left ventricular hypertrophy (LVH) on echocardiography in patients with CKD compared to healthy controls and a significant independent association between LVH and albuminuria (P = 0.002) (*44*). In a study of 1818 patients with acute decompensated heart failure, Wang *et al*. found that patients with albuminuria had a significantly increased risk of all-cause death or heart transplantation/LVAD implantation than those without albuminuria, even after adjustment for other significant clinical factors (age, history of arterial hypertension, presence of atrial fibrillation/flutter, New York Heart Association (NYHA) class, heart rate, systolic blood pressure, body mass index (BMI), haemoglobin, serum albumin, serum creatinine, eGFR, N-terminal pro-brain natriuretic peptide (NT‐proBNP), left ventricular diastolic dysfunction, left ventricular ejection fraction (LVEF) and prescription of angiotensin converting enzyme inhibitors/angiotensin receptor blockers, β‐blockers or diuretics) (HR = 1.28, 95%CI 1.09-1.50, P = 0.003). The risk of the adverse events listed was proportional with the increase in the degree of albuminuria (P = 0.004) (*45*). Kato *et al*. studied the association between serum albumin concentrations and 1-year adverse outcomes in acute decompensated heart failure patients and found that increased serum albumin concentrations were significantly associated with a lower risk of death or hospitalization due to heart failure (HR = 0.78, 95% CI 0.69-0.90, P < 0.001) after adjusting for baseline albumin concentrations, anaemia, age, eGFR, BMI, NYHA and history of diabetes mellitus (*46*). A study by Alatas *et al*. demonstrated that the microalbuminuria was a predictor of in-hospital mortality in acute heart failure patients with restricted and mid-range ejection fractions, but not in patients with preserved ejection fraction (*47*).

To summarize, albuminuria, regardless of the degree of its severity (micro or macro) is an important predictor of adverse short- and long-term outcomes in patients with both acute and chronic heart failure. Given its general availability and low cost as a laboratory test, it should be utilized whenever possible in order to stratify heart failure patients into groups based on risk of adverse outcomes and therefore guide their in-hospital and out-of-hospital management and long-term plans of care.

## Biomarkers of cardiac injury

### Troponin

Troponins are a family of proteins that play an important part in the regulation of skeletal and cardiac muscle contractility mechanism. There are three isoforms of troponin: C, T and I. Troponin C is found in both cardiac and skeletal muscle, but troponin T and I are highly specific for cardiac muscle tissue, therefore being suitable markers of myocardial injury (*48*). Immunohistological evidence has demonstrated that extent of myocardial injury is positively correlated with serum troponin concentrations (*49*). Serum troponin concentrations may also increase in the context of decreased respiratory function and intense physical exercise without a myocardial tissue injury (*50*). In patients with CKD, there is a persistent elevation of serum troponin concentration, regardless of the existence of myocardial injury. This phenomenon is explained by both the lowered rate of serum troponin clearance (troponin is eliminated by the kidneys) and a higher rate of troponin release from cardiomyocytes due to subclinical injury (*i.e.* uremic toxicity, hypertensive heart disease) (*51*). A recent retrospective study conducted on Chronic Renal Insufficiency Cohort (CRIC) participants analysed ambulatory CKD patients’ serum high sensitivity troponin T (hsTnT) concentrations in order to test the reliability of the conventional upper reference limits (URLs) for hsTnT in CKD patients. The authors found that among 2312 patients with CKD, 43% had a resting hsTnT concentration above the conventional URL. This finding was even more pronounced in patients with advanced renal failure (CKD grade IV, eGFR < 30 mL/min/1.73m^2^), 68% of which had a resting hsTnT concentration above the conventional URL. Further data analysis provided a model demonstrating that, in patients with CKD, the threshold for the 99^th^ percentile of serum hsTnT concentrations increases by 44% for every eGFR decrease of 15 mL/min/1.73m^2^ (*52*). However, despite those findings, elevated hsTnT serum concentrations have been demonstrated as a significant predictor of 2-year mortality (AUC 0.69, 74% sensitivity, 63% specificity, 90% negative predictive value) in patients with acute chest pain and impaired renal function (defined as eGFR < 60 mL/min/m^2^) (*53*). Another study investigating the predictive value of high sensitivity troponin I (hsTnI) serum concentrations in patients with CKD had similar findings. Although the diagnostic accuracy of hsTnI concentrations was lower in CKD patients compared to patients with preserved renal function (positive predictive value 50% *vs*. 63%, specificity 71% *vs.* 92%), CKD patients with hsTnI concentrations above the 99^th^ percentile had a significantly higher risk of myocardial infarction or cardiac death at 1-year follow up compared to patients with elevated hsTnI concentrations and preserved renal function (24% *vs.* 10%, HR = 2.19, 95%CI 1.54-3.11) (*54*). There is evidence that, with several modifications, the diagnostic accuracy of hsTnT concentrations for acute myocardial injury in patients with CKD can be significantly improved – Alushi *et al*. found that, by using hsTnT cut-offs 4 times greater than the conventional ones, the specificity rose from 10% to 65%, but the sensitivity decreased from 98% to 83%. This was mitigated by designing a model combining the baseline hsTnT concentration with an absolute change in hsTnT concentration 3 hours following the index measurement. Such a model yielded improved diagnostic accuracy – 98% sensitivity, 55% specificity, 93% positive predictive value, 86% negative predictive value (*55*). The predictive value of troponin in CRS is limited and debatable. Ledwoch *et al*. found that in patients with acute heart failure and impaired renal function (defined as eGFR < 45 mL/min/1.73m^2^), hsTnT had a significantly lower predictive accuracy for 30-day mortality compared to patients with acute heart failure and preserved renal function (AUC 0.63 *vs*. 0.74, P = 0.049) (*56*). He *et al*. evaluated the diagnostic accuracy of cardiac troponin I (cTnI) in predicting the development of type I CRS in patients with acute myocardial infarction. The AUC for cTnI was 0.76, however, when cTnI was combined with NT-proBNP, baseline eGFR and white blood cell count in a statistical model, the AUC rose to 0.92, indicating that other biomarkers can supplement and increase the predictive and diagnostic value of cTnI for CRS when combined (*57*).

Although serum troponin concentrations are elevated at baseline in patients with CKD due to impaired renal clearance, making the usage of regular cut-off values ineffective and inaccurate, it would seem that any serial elevation of serum troponin concentration in patients with chest pain, regardless of renal function, is a predictor of adverse long-term outcomes. Therefore, an elevation in serial troponin concentrations in chest pain patients should alarm the clinician and place the patient into a higher risk category.

While serum troponin by itself is not a great predictor of the development of type I CRS following an acute myocardial infarction, if combined with indicators of cardiac and renal function (eGFR, NT-proBNP), it can predict complications with an excellent diagnostic accuracy.

### N-terminal pro-brain natriuretic peptide

N-terminal pro-brain natriuretic peptide is a prohormone of brain natriuretic peptide - a peptide secreted by cardiomyocytes as a response to ventricular stretching caused by an increase in circulatory volume. As a surrogate for volume overload, NT-proBNP has been extensively researched in acute heart failure, acute kidney injury and CRS. Yamashita *et al*. studied several different cardiac biomarkers in patients hospitalized through the emergency department for cardiac emergencies and found that patients with a serum NT-proBNP < 689 pg/mL had significantly higher rates of survival than those with a serum NT-proBNP > 689 pg/mL, regardless of the primary cardiac diagnosis (*58*). Zhang *et al.* studied the role of NT-proBNP in predicting type I CRS in patients with acute myocardial infarction and found that elevated NT-proBNP serum concentrations independently predicted type I CRS with an AUC of 0.72 (95%CI 0.78-0.85). When NT-proBNP was combined with eGFR and high sensitivity C-reactive protein (hsCRP), the AUC rose to 0.86 (95%CI 0.83-0.89) (*59*). N-terminal pro-brain natriuretic peptide as a biomarker of fluid overload is especially valuable in acute heart failure patients and has even been shown to predict renal function decline. McCallum *et al*. analysed data from the Efficacy of Vasopressin Antagonism in Heart Failure Outcome Study With Tolvaptan (EVEREST) trial and found that patients suffering from heart failure with reduced ejection fraction (HFrEF) who had elevated NT-proBNP serum concentrations were at significantly higher risk of developing a > 40% eGFR decline (HR = 2.62, 95%CI 1.62-4.23) (*60*). Another study by McCallum *et al.* analysed patient data from the Ultrafiltration in Decompensated Heart Failure with Cardiorenal Syndrome Study (CARRESS) and Diuretic Optimization Strategies Evaluation (DOSE) trials. In this patient pool, consisting of patients with acute decompensated heart failure, the authors found that an in-hospital decline in eGFR was not significantly associated with an increased risk of death or rehospitalization, while an in-hospital decline in NT-proBNP was significantly associated with a lower risk of death or rehospitalization. The authors also found that a decline in eGFR was associated with decreased risk of death or rehospitalization if the NT-proBNP declined along with the eGFR (*61*). De la Espriella *et al*. evaluated the prognostic value of NT-proBNP in acute heart failure patients based on renal function and found that while serum NT-proBNP concentration was positively and linearly associated with mortality in this patient group, its predictive value significantly decreased in patients with eGFR < 45 mL/min/1.73m^2^ (*62*). N-terminal pro-brain natriuretic peptide has also been researched as a urinary biomarker – Zhao *et al*. investigated the predictive value of urinary NT-proBNP (uNT-proBNP) for the development of type I CRS in patients with acute decompensated heart failure. They found that uNT-proBNP was a significant and reliable predictor of type I CRS development, with an AUC of 0.93 (95%CI 0.87-0.97) (*63*).

It can be inferred from the findings of the studies cited in this paragraph that while NT-proBNP has a decent diagnostic accuracy for CRS, especially if combined with markers of inflammation and indicators of renal function, its greatest value lies in the fact that it can be utilized in order to guide pharmacologic therapy. As demonstrated by McCallum *et al.*, NT-proBNP is a significantly more reliable indicator of the effectiveness of diuretic therapy than eGFR (*65*). Furthermore, relying on eGFR exclusively for the titration of diuretic therapy in fluid overloaded CRS patients would lead to premature cessation or de-escalation of treatment due to observed (falsely) worsened renal function. Instead, diuretic therapy should be directed and guided by NT-proBNP measurements and clinical findings (physical examination and ultrasonography), given that NT-proBNP is the only reliable predictor of adverse short- and long-term outcomes in this patient population.

### Fibrosis index 4 and 5

Fibrosis index 4 (FIB-4) is a simple score for estimating the degree of hepatic fibrosis, that is calculated from patient age, platelet count, aspartate aminotransferase (AST) and alanine aminotransferase (ALT) serum activities (*64*). Researchers have found predictive value in FIB-4 for the prognosis of long-term outcomes in hospitalized heart failure patients. Nakashima *et al*. studied patients with acute decompensated heart failure with preserved ejection fraction and found that a high FIB-4 before hospital discharge was significantly associated with major adverse cardiovascular events (MACE) (HR = 1.27, 95%CI 1.05-1.53) after adjusting for patient sex, serum haemoglobin concentration and serum creatinine concentration. They found that the cut-off for increased risk of MACE was a FIB-4 of ≥ 3.11 (*65*). Okamoto *et al*. also found that in patients with subclinical heart failure with preserved ejection fraction, FIB-4 is an independent predictor of all-cause mortality and hospitalization for heart failure (HR = 1.31, 95%CI 1.14-1.50, P < 0.001) (*66*). Ewid *et al*. found that an AST/ALT ratio ≥ 1 was associated with and predictive of a LVEF < 30% on echocardiography, with an AUC of 0.64 (95%CI 0.54-0.73) (P < 0.05), 44% sensitivity and 81% specificity (*67*). Fibrosis index 5 (FIB-5), another score for estimating hepatic fibrosis, was studied by Maeda *et al.* Their research showed that FIB-5, calculated from serum albumin, AST, ALT, alkaline phosphatase (ALP) serum activities and platelet count was superior to FIB-4 at predicting death or hospital readmission for heart failure in patients hospitalized with acute decompensated heart failure, after adjusting for significant clinical factors. Another interesting finding is that, while high FIB-4 scores before hospital discharge are associated with an increased risk of MACE, patients with high FIB-5 scores have a lowest risk of death or hospital readmission, while those with low FIB-5 scores have a significantly increased risk of adverse events (*68*).

Due to primarily being markers of hepatic injury and fibrosis, FIB-4 and FIB-5 scores have not been extensively researched in the context of heart failure. However, the available literature suggests a potential value of those scores in stratifying hospitalized heart failure patients based on the risk of long-term adverse outcomes. Since they are simple scores utilizing routinely measured laboratory biomarkers, they should be used more often in heart failure patients prior to hospital discharge along with other predictors of long-term outcomes in order to risk stratify patients and formulate an optimal long-term care strategy tailored to individual patient risk.

## Conclusions

With the advancements made in the field medical biochemistry, novel biochemical markers of renal and cardiac function are emerging as a possible replacement or supplement to existing ones. At the same time, the utility and roles of traditional cardiorenal biomarkers are being re-examined in order to maximize their diagnostic and prognostic utility in the appropriate patient populations and disease states. While cystatin C is gaining popularity as a marker of glomerular function and is possibly more accurate than serum creatinine, creatinine measurements are still the mainstay of glomerular function estimation, being recommended as the basis of calculations endorsed by current guidelines. Neutrophil gelatinase-associated lipocalin as a marker of early, subclinical AKI may serve as a tool for timely detection of renal injury and can indicate the need for initiation of specific protective and curative measures. N-terminal pro-brain natriuretic peptide is the most reliable marker of response to treatment in type I CRS and should be used to guide the duration and intensity of diuretic therapy. Troponins are valuable predictors of adverse long-term outcomes in patients with acute myocardial infarction and AKI or CKD. Albuminuria can be used as a reliable predictor of adverse outcomes in patients with CRS and should be measured during the patients’ hospital stay, before discharge and on follow-up examinations. Finally, indicators of hepatic damage or fibrosis, like FIB-4 and FIB-5, might be a reliable prognostic tool of long term adverse outcomes for heart failure patients being discharged from the hospital. Physicians managing patients with CRS should be acquainted with the diagnostic accuracy and specific features of different biomarkers in order to gain more value from their use, predict patient important outcomes more accurately and provide goal-directed management and therapy.

## Data Availability

No data was generated during this study.

## References

[1] National Heart, Lung, and Blood Institute. Cardio-Renal Connections in Heart Failure and Cardiovascular Disease. Available from: https://www.nhlbi.nih.gov/events/2004/cardio-renal-connections-heart-failure-and-cardiovascular-disease. Accessed February 16th 2023.

[2] Ronco C McCullough P Anker SD Anand I Aspromonte N Bagshaw SM Cardio-renal syndromes: report from the consensus conference of the acute dialysis quality initiative. Eur Heart J. 2010;31:703–11. 10.1093/eurheartj/ehp507 20037146 PMC2838681

[3] Hatamizadeh P Fonarow GC Budoff MJ Darabian S Kovesdy CP Kalantar-Zadeh K. Cardiorenal syndrome: pathophysiology and potential targets for clinical management. Nat Rev Nephrol. 2013;9:99–111. 10.1038/nrneph.2012.279 23247571

[4] Mehta RL Kellum JA Shah SV Molitoris BA Ronco C Warnock DG Acute Kidney Injury Network: report of an initiative to improve outcomes in acute kidney injury. Crit Care. 2007;11:R31. 10.1186/cc5713 17331245 PMC2206446

[5] Bellomo R Ronco C Kellum JA Mehta RL Palevsky P. Acute renal failure: definition, outcome measures, animal models, fluid therapy and information technology needs: the Second International Consensus Conference of the Acute Dialysis Quality Initiative (ADQI) Group. Crit Care. 2004;8:R204–12. 10.1186/cc2872 15312219 PMC522841

[6] Kidney Disease: Improving Global Outcomes (KDIGO) Acute Kidney Injury Work Group. KDIGO clinical practice guideline for acute kidney injury. Kidney Int. 2012;4:1–138.

[7] Ahmad T Jackson K Rao VS Tang WHW Brisco-Bacik MA Chen HH Worsening renal function in patients with acute heart failure undergoing aggressive diuresis is not associated with tubular injury. Circulation. 2018;137:2016–28. 10.1161/CIRCULATIONAHA.117.030112 29352071 PMC6066176

[8] Wyss M Kaddurah-Daouk R. Creatine and creatinine metabolism. Physiol Rev. 2000;80:1107–213. 10.1152/physrev.2000.80.3.1107 10893433

[9] Andreev E Koopman M Arisz L. A rise in plasma creatinine that is not a sign of renal failure: which drugs can be responsible? J Intern Med. 1999;246:247–52. 10.1046/j.1365-2796.1999.00515.x 10475992

[10] Dunn SR Gabuzda GM Superdock KR Kolecki RS Schaedler RW Simenhoff ML. Induction of creatininase activity in chronic renal failure: timing of creatinine degradation and effect of antibiotics. Am J Kidney Dis. 1997;29:72–7. 10.1016/S0272-6386(97)90010-X 9002532

[11] Bouchard J Macedo E Soroko S Chertow GM Himmelfarb J Ikizler TA Comparison of methods for estimating glomerular filtration rate in critically ill patients with acute kidney injury. Nephrol Dial Transplant. 2010;25:102–7. 10.1093/ndt/gfp392 19679558 PMC2910324

[12] Raimundo M Crichton S Martin JR Syed Y Varrier M Wyncoll D Increased Fluid Administration After Early Acute Kidney Injury is Associated with Less Renal Recovery. Shock. 2015;44:431–7. 10.1097/SHK.0000000000000453 26263435

[13] Niemantsverdriet MSA Pieters TT Hoefer IE Verhaar MC Joles JA van Solinge WW GFR estimation is complicated by a high incidence of non-steady-state serum creatinine concentrations at the emergency department. PLoS One. 2021;16:e0261977. 10.1371/journal.pone.0261977 34965267 PMC8716053

[14] Brisco MA Zile MR Hanberg JS Wilson FP Parikh CR Coca SG Relevance of Changes in Serum Creatinine During a Heart Failure Trial of Decongestive Strategies: Insights From the DOSE Trial. J Card Fail. 2016;22:753–60. 10.1016/j.cardfail.2016.06.423 27374839 PMC5435117

[15] Phan Thai H Hoang Bui B Hoang Anh T Huynh Van M. Value of Plasma NGAL and Creatinine on First Day of Admission in the Diagnosis of Cardiorenal Syndrome Type 1. Cardiol Res Pract. 2020;2020:2789410. 10.1155/2020/2789410 33083054 PMC7559494

[16] Delanaye P Schaeffner E Cozzolino M Langlois M Plebani M Ozben T The new, race-free, Chronic Kidney Disease Epidemiology Consortium (CKD-EPI) equation to estimate glomerular filtration rate: is it applicable in Europe? A position statement by the European Federation of Clinical Chemistry and Laboratory Medicine (EFLM). Clin Chem Lab Med. 2022;61:44–7. 10.1515/cclm-2022-0928 36279207

[17] Abrahamson M Alvarez-Fernandez M Nathanson CM. Cystatins. Biochem Soc Symp. 2003;70:179–99. 10.1042/bss0700179 14587292

[18] Filler G Bökenkamp A Hofmann W Le Bricon T Martínez-Brú C Grubb A. Cystatin C as a marker of GFR--history, indications, and future research. Clin Biochem. 2005;38:1–8. 10.1016/j.clinbiochem.2004.09.025 15607309

[19] Chen S Tang Y Zhou X. Cystatin C for predicting all-cause mortality and rehospitalization in patients with heart failure: a meta-analysis. Biosci Rep. 2019;39:BSR20181761. 10.1042/BSR20181761 30643006 PMC6361773

[20] Pinsino A Mondellini GM Royzman EA Hoffman KL D’Angelo D Mabasa M Cystatin C- Versus Creatinine-Based Assessment of Renal Function and Prediction of Early Outcomes Among Patients With a Left Ventricular Assist Device. Circ Heart Fail. 2020;13:e006326. 10.1161/CIRCHEARTFAILURE.119.006326 31959016

[21] Zhang Y Yang S Chen J Zhang Z He P Zhou C Associations of serum cystatin C and its change with new-onset cardiovascular disease in Chinese general population. Nutr Metab Cardiovasc Dis. 2022;32:1963–71. 10.1016/j.numecd.2022.05.016 35738955

[22] Ruan ZB Zhu L Yin YG Chen GC Cystatin C. N-terminal probrain natriuretic peptides and outcomes in acute heart failure with acute kidney injury in a 12-month follow-up: Insights into the cardiorenal syndrome. J Res Med Sci. 2014;19:404–9. 25097621 PMC4116570

[23] Rafouli-Stergiou P Parissis J Farmakis D Bistola V Nikolaou M Vasiliadis K Prognostic value of in-hospital change in cystatin C in patients with acutely decompensated heart failure and renal dysfunction. Int J Cardiol. 2015;182:74–6. 10.1016/j.ijcard.2014.12.135 25576725

[24] Yong Z Pei X Zhu B Yuan H Zhao W. Predictive value of serum cystatin C for acute kidney injury in adults: a meta-analysis of prospective cohort trials. Sci Rep. 2017;7:41012. 10.1038/srep41012 28112204 PMC5253621

[25] Shardlow A McIntyre NJ Fraser SDS Roderick P Raftery J Fluck RJ The clinical utility and cost impact of cystatin C measurement in the diagnosis and management of chronic kidney disease: A primary care cohort study. PLoS Med. 2017;14:e1002400. 10.1371/journal.pmed.1002400 29016597 PMC5634538

[26] Shlipak MG Matsushita K Ärnlöv J Inker LA Katz R Polkinghorne KR Cystatin C versus creatinine in determining risk based on kidney function. N Engl J Med. 2013;369:932–43. 10.1056/NEJMoa1214234 24004120 PMC3993094

[27] Makris K Rizos D Kafkas N Haliassos A. Neurophil gelatinase-associated lipocalin as a new biomarker in laboratory medicine. Clin Chem Lab Med. 2012;50:1519–32. 10.1515/cclm-2012-0227 23104835

[28] Mishra J Ma Q Prada A Mitsnefes M Zahedi K Yang J Identification of neutrophil gelatinase-associated lipocalin as a novel early urinary biomarker for ischemic renal injury. J Am Soc Nephrol. 2003;14:2534–43. 10.1097/01.ASN.0000088027.54400.C6 14514731

[29] Song X Cai D Zhang B. Clinical values of serum NGAL combined with NT-proBNP in the early prognosis of type 1 cardiorenal syndrome. Am J Transl Res. 2021;13:3363–8. 34017511 PMC8129259

[30] Alvelos M Pimentel R Pinho E Gomes A Lourenço P Teles MJ Neutrophil gelatinase-associated lipocalin in the diagnosis of type 1 cardio-renal syndrome in the general ward. Clin J Am Soc Nephrol. 2011;6:476–81. 10.2215/CJN.06140710 21115620 PMC3082403

[31] Ferrari F Scalzotto E Esposito P Samoni S Mistrorigo F Rizo Topete LM Neutrophil gelatinase-associated lipocalin does not predict acute kidney injury in heart failure. World J Clin Cases. 2020;8:1600–7. 10.12998/wjcc.v8.i9.1600 32432138 PMC7211536

[32] Maisel AS Wettersten N van Veldhuisen DJ Mueller C Filippatos G Nowak R Neutrophil Gelatinase-Associated Lipocalin for Acute Kidney Injury During Acute Heart Failure Hospitalizations: The AKINESIS Study. J Am Coll Cardiol. 2016;68:1420–31. 10.1016/j.jacc.2016.06.055 27659464

[33] Soyler C Tanriover MD Ascioglu S Aksu NM Arici M. Urine neutrophil gelatinase-associated lipocalin levels predict acute kidney injury in acute decompensated heart failure patients. Ren Fail. 2015;37:772–6. 10.3109/0886022X.2015.1033324 25869054

[34] Nasonova SN Zhirov IV Ledyakhova MV Sharf TV Bosykh EG Masenko VP Early diagnosis of acute renal injury in patients with acute decompensation of chronic heart failure. Ter Arkh. 2019;91:67–73. 10.26442/00403660.2019.04.000168 31094479

[35] Palazzuoli A Ruocco G Beltrami M Franci B Pellegrini M Lucani B Admission plasma neutrophil gelatinase associated lipocalin (NGAL) predicts worsening renal function during hospitalization and post discharge outcome in patients with acute heart failure. Acute Card Care. 2014;16:93–101. 10.3109/17482941.2014.911915 24836558

[36] Haase M Devarajan P Haase-Fielitz A Bellomo R Cruz DN Wagener G The outcome of neutrophil gelatinase-associated lipocalin-positive subclinical acute kidney injury: a multicenter pooled analysis of prospective studies. J Am Coll Cardiol. 2011;57:1752–61. 10.1016/j.jacc.2010.11.051 21511111 PMC4866647

[37] Kim H Hur M Cruz DN Moon HW Yun YM. Plasma neutrophil gelatinase-associated lipocalin as a biomarker for acute kidney injury in critically ill patients with suspected sepsis. Clin Biochem. 2013;46:1414–8. 10.1016/j.clinbiochem.2013.05.069 23747960

[38] Stevens PE Levin A. Evaluation and management of chronic kidney disease: synopsis of the kidney disease: improving global outcomes 2012 clinical practice guideline. Ann Intern Med. 2013;158:825–30. 10.7326/0003-4819-158-11-201306040-00007 23732715

[39] Matsushita K van der Velde M Astor BC Woodward M Levey AS de Jong PE Association of estimated glomerular filtration rate and albuminuria with all-cause and cardiovascular mortality in general population cohorts: a collaborative meta-analysis. Lancet. 2010;375:2073–81. 10.1016/S0140-6736(10)60674-5 20483451 PMC3993088

[40] Astor BC Matsushita K Gansevoort RT van der Velde M Woodward M Levey AS Lower estimated glomerular filtration rate and higher albuminuria are associated with mortality and end-stage renal disease. A collaborative meta-analysis of kidney disease population cohorts. Kidney Int. 2011;79:1331–40. 10.1038/ki.2010.550 21289598 PMC3917543

[41] Rifkin DE Katz R Chonchol M Fried LF Cao J de Boer IH Albuminuria, impaired kidney function and cardiovascular outcomes or mortality in the elderly. Nephrol Dial Transplant. 2010;25:1560–7. 10.1093/ndt/gfp646 20008829 PMC3307251

[42] Funahashi Y Ikeda M Wakasaki R Chowdhury S Groat T Zeppenfeld D Renal injury in cardiorenal syndrome type 1 is mediated by albumin. Physiol Rep. 2022;10:e15173. 10.14814/phy2.15173 35150207 PMC8838648

[43] Jackson CE Solomon SD Gerstein HC Zetterstrand S Olofsson B Michelson EL Albuminuria in chronic heart failure: prevalence and prognostic importance. Lancet. 2009;374:543–50. 10.1016/S0140-6736(09)61378-7 19683640

[44] Landler NE Olsen FJ Christensen J Bro S Feldt-Rasmussen BO Hansen D Associations Between Albuminuria, Estimated GFR and Cardiac Phenotype in a Cohort with Chronic Kidney Disease: The CPH-CKD ECHO Study. J Card Fail. 2022;28:1615–27. 10.1016/j.cardfail.2022.09.002 36126901

[45] Wang Y Zhao X Zhai M Fan C Huang Y Zhou Q Elevated urinary albumin concentration predicts worse clinical outcomes in hospitalized acute decompensated heart failure patients. ESC Heart Fail. 2021;8:3037–48. 10.1002/ehf2.13399 34008352 PMC8318403

[46] Kato T Yaku H Morimoto T Inuzuka Y Tamaki Y Ozasa N Association of an increase in serum albumin levels with positive 1-year outcomes in acute decompensated heart failure: A cohort study. PLoS One. 2020;15:e0243818. 10.1371/journal.pone.0243818 33370299 PMC7769473

[47] Alataş ÖD Biteker M Demir A Yıldırım B Acar E Gökçek K Microalbuminuria and its Prognostic Significance in Patients with Acute Heart Failure with Preserved, Mid-Range, and Reduced Ejection Fraction. Arq Bras Cardiol. 2022;118:703–9. 35137781 10.36660/abc.20201144 PMC9007018

[48] Ooi DS Isotalo PA Veinot JP. Correlation of antemortem serum creatine kinase, creatine kinase-MB, troponin I, and troponin T with cardiac pathology. Clin Chem. 2000;46:338–44. 10.1093/clinchem/46.3.338 10702520

[49] Fishbein MC Wang T Matijasevic M Hong L Apple FS. Myocardial tissue troponins T and I. An immunohistochemical study in experimental models of myocardial ischemia. Cardiovasc Pathol. 2003;12:65–71. 10.1016/S1054-8807(02)00188-6 12684160

[50] Agewall S Giannitsis E Jernberg T Katus H. Troponin elevation in coronary vs. non-coronary disease. Eur Heart J. 2011;32:404–11. 10.1093/eurheartj/ehq456 21169615

[51] Parikh RH Seliger SL deFilippi CR. Use and interpretation of high sensitivity cardiac troponins in patients with chronic kidney disease with and without acute myocardial infarction. Clin Biochem. 2015;48:247–53. 10.1016/j.clinbiochem.2015.01.004 25617663

[52] Bansal N Zelnick LR Ballantyne CM Chaves PHM Christenson RH Coresh J Upper Reference Limits for High-Sensitivity Cardiac Troponin T and N-Terminal Fragment of the Prohormone Brain Natriuretic Peptide in Patients With CKD. Am J Kidney Dis. 2022;79:383–92. 10.1053/j.ajkd.2021.06.017 34293394 PMC8766621

[53] Haaf P Reichlin T Twerenbold R Hoeller R Rubini Gimenez M Zellweger C Risk stratification in patients with acute chest pain using three high-sensitivity cardiac troponin assays. Eur Heart J. 2014;35:365–75. 10.1093/eurheartj/eht218 23821402

[54] Miller-Hodges E Anand A Shah ASV Chapman AR Gallacher P Lee KK High-Sensitivity Cardiac Troponin and the Risk Stratification of Patients With Renal Impairment Presenting With Suspected Acute Coronary Syndrome. Circulation. 2018;137:425–35. 10.1161/CIRCULATIONAHA.117.030320 28978551 PMC5793996

[55] Alushi B Jost-Brinkmann F Kastrati A Cassese S Fusaro M Stangl K High-Sensitivity Cardiac Troponin T in Patients with Severe Chronic Kidney Disease and Suspected Acute Coronary Syndrome. J Clin Med. 2021;10:4216. 10.3390/jcm10184216 34575325 PMC8471888

[56] Ledwoch J Krauth A Kraxenberger J Schneider A Leidgschwendner K Schneider V Accuracy of high-sensitive troponin depending on renal function for clinical outcome prediction in patients with acute heart failure. Heart Vessels. 2022;37:69–76. 10.1007/s00380-021-01890-3 34152442 PMC8732937

[57] He CH Liu JW Zhu ZH Pan HW Zheng ZF He J Establishment and validation of a new predictive equation with multiple risk factors for the development of cardiorenal syndrome type 1 in patients with acute myocardial infarction. Zhonghua Xin Xue Guan Bing Za Zhi. 2021;49:802–8. [in Chinese] 34404190 10.3760/cma.j.cn112148-20201118-00916

[58] Yamashita T Seino Y Ogawa A Ogata K Fukushima M Tanaka K N-terminal pro-BNP is a novel biomarker for integrated cardio-renal burden and early risk stratification in patients admitted for cardiac emergency. J Cardiol. 2010;55:377–83. 10.1016/j.jjcc.2010.01.008 20350516

[59] Zhang DQ Li HW Chen HP Ma Q Chen H Xing YL Combination of Amino-Terminal Pro- BNP, Estimated GFR, and High-Sensitivity CRP for Predicting Cardiorenal Syndrome Type 1 in Acute Myocardial Infarction Patients. J Am Heart Assoc. 2018;7:e009162. 10.1161/JAHA.118.009162 30371311 PMC6404877

[60] McCallum W Tighiouart H Testani JM Griffin M Konstam MA Udelson JE Association of Volume Overload With Kidney Function Outcomes Among Patients With Heart Failure With Reduced Ejection Fraction. Kidney Int Rep. 2020;5:1661–9. 10.1016/j.ekir.2020.07.015 33102958 PMC7569703

[61] McCallum W Tighiouart H Kiernan MS Huggins GS Sarnak MJ. Relation of Kidney Function Decline and NT-proBNP With Risk of Mortality and Readmission in Acute Decompensated Heart Failure. Am J Med. 2020;133:115–22.e2. 10.1016/j.amjmed.2019.05.047 31247182 PMC7373496

[62] de la Espriella R Bayés-Genís A Llàcer P Palau P Miñana G Santas E Prognostic value of NT-proBNP and CA125 across glomerular filtration rate categories in acute heart failure. Eur J Intern Med. 2022;95:67–73. 10.1016/j.ejim.2021.08.024 34507853

[63] Zhao HL Hu HJ Zhao XJ Chi WW Liu DM Wang Q Urine N-terminal pro-B-type natriuretic peptide and plasma proenkephalin are promising biomarkers for early diagnosis of cardiorenal syndrome type 1 in acute decompensated heart failure: a prospective, double-center, observational study in real-world. Ren Fail. 2022;44:1486–97. 10.1080/0886022X.2022.2114367 36000917 PMC9423828

[64] Lee J Vali Y Boursier J Spijker R Anstee QM Bossuyt PM Prognostic accuracy of FIB-4, NAFLD fibrosis score and APRI for NAFLD-related events: A systematic review. Liver Int. 2021;41:261–70. 10.1111/liv.14669 32946642 PMC7898346

[65] Nakashima M Sakuragi S Miyoshi T Takayama S Kawaguchi T Kodera N Fibrosis-4 index reflects right ventricular function and prognosis in heart failure with preserved ejection fraction. ESC Heart Fail. 2021;8:2240–7. 10.1002/ehf2.13317 33760403 PMC8120399

[66] Ewid M Sherif H Allihimy AS Alharbi SA Aldrewesh DA Alkuraydis SA AST/ALT ratio predicts the functional severity of chronic heart failure with reduced left ventricular ejection fraction. BMC Res Notes. 2020;13:178. 10.1186/s13104-020-05031-3 32209113 PMC7092498

[67] Maeda D Kanzaki Y Sakane K Tsuda K Akamatsu K Hourai R Prognostic value of the liver fibrosis marker fibrosis-5 index in patients with acute heart failure. ESC Heart Fail. 2022;9:1380–7. 10.1002/ehf2.13829 35119215 PMC8934979

[68] Okamoto C Tsukamoto O Hasegawa T Hitsumoto T Matsuoka K Amaki M Candidate Screening for Heart Failure With Preserved Ejection Fraction Clinic by Fib-4 Index From Subclinical Subjects. Gastro Hep Adv. 2023;2:170–81. 10.1016/j.gastha.2022.09.005 PMC11307393 39132617

